# Adjustable-Volume Prosthetic Sockets: Market Overview and Value Propositions

**DOI:** 10.33137/cpoj.v4i2.35208

**Published:** 2021-09-21

**Authors:** TD Klenow, J. Schulz

**Affiliations:** 1 Martin Bionics Clinical Care, Fort Myers, Florida, USA.; 2 Martin Bionics Innovations, Oklahoma City, Oklahoma, USA.

**Keywords:** Innovation, Interface, Amputation, Limb Loss, Rehabilitation, Modular, Prosthetic Sockets, Prosthetics, Medical Equipment

## Abstract

The prosthetic socket is commonly considered to be the most important part of the prosthesis and lack of fit can lead to skin breakdown, reduction in wear, reduction in activity, and consequential deleterious health effects. Furthermore, approximately 90% of amputations are due to a vascular etiology, which affect fluid retention regularity, and even small limb volume fluctuations can lead to lack of fit. Adjustability in the socket volume has been suggested as a potential solution to common fit issues but has lacked market penetration mostly due to lack of reimbursement. Despite this there are several adjustable-volume sockets emerging on the market today including prefabricated, modular, custom with adjustable-volume component, custom with adjustable-volume feature, and adjustable-hybrid sockets. Prefabricated sockets are mass produced in common sizes and fit directly to the patient by a prosthetist using pad kits, BOA dials, or straps. Modular sockets are assembled to a patient or model with panels or struts attached to an adjustable base. Custom sockets with adjustable-volume elements are traditionally-fabricated sockets made to a model of a patient's limb with a volume-adjustable component added or volume-adjustable feature built in. Custom-hybrid sockets are made custom to a model of the patient's limb and incorporate several aspects of the previous socket types and include some radically-unique design aspects which cannot be limited to one category. These adjustable-volume sockets offer several advantages to traditional rigid-volume sockets for the patient, prosthetist, and providing clinic. The micro-adjustability for the patient allows them to alter fit without removing the socket, maintaining a more intimate fit throughout the day than traditional sockets. The macro-adjustability for the prosthetist allows for increased options for fit customization including the ability to reverse or undo changes without necessarily re-making the socket. This allows for the most optimal fit for the patient. Adjustable volume also present efficiencies in the fitting process by simplifying or eliminating steps including residual limb shape capture, form modification, diagnostic fabrication, iterative alteration, and definitive fabrication with the different socket types affecting different steps. Due to these factors, adjustable-volume sockets have disrupted the market to the point where reimbursement reform is needed including additional L-codes in the United States and fee-for-service or fee-for-outcome associated with prosthetic follow-up care. Prosthetic care should also be separated from durable medical equipment to allow for alternative reimbursement models. As reimbursement adapts prosthetists must adapt correspondingly to differentiate their skillset from other allied health providers including incorporating more objective methods to show superior care outcomes. This adaptation should include a continued push for state and municipal licensure of prosthetists.

## INTRODUCTION

The human-device interface, referred to clinically as the socket, is commonly considered to be the most important part of a prosthesis.^1–6^ It is also the most problematic, however, as lack of socket fit is a commonly reported issue among end-users.^5^ Since the socket is the only custom-fabricated part of the prosthesis, it's replacement represents the largest time commitment to the patient.^1,7^ Approximately 90% of amputations occur secondary to diabetes and vascular disorders which leads to complex clinical presentations in much of the population.^4,8^ Vascular compromise amplifies fluid retention difficulties and even small changes in limb volume can lead to socket fit issues.^9^

Lack of socket fit can lead to pain, discomfort, skin irritation and breakdown, subsequent prosthetic abandonment, and therefore activity reduction, reduced social participation, psychosocial problems, and deleterious health effects due to inactivity.^2–8^ Further, only 50% of individuals with amputation receive a prosthesis initially and 11–22% of patients abandon their prosthesis at one year.^10^ Re-amputation and mortality rates surrounding amputation also remain remarkably high thereafter.^4,11^

These pervasive issues, described above, among the patient population have stimulated several innovations in interface design over the past decade.^5,9^ A notable innovation is the introduction of additive manufacturing, or 3D-printing, to prosthetic socket fabrication.^12^ 3D-printed sockets offer an improved array of material selection, textured finishes, and elasticity. They also offer new design elements as wall thicknesses can be increased in areas where more structural support is required and decreased where more flexibility is desired. With all the benefits inherent in 3D-printed sockets, they still rely on traditional methods to accommodate residual limb volume changes.^8,9^ Therefore, additive manufacturing alone does not adequately address residual limb fluid dynamics and associated socket fit issues over time.

Adjustable sockets are designed as an innovation to provide on-demand micro-adjustability to the patient and/or macro-adjustability to the prosthetist. Micro-adjustability is a socket feature which allows the patient to accommodate diurnal residual limb volume fluctuation without needing to remove the socket to implement a traditional volume management strategy. Macro-adjustability is a socket feature which allows the prosthetist to accommodate large physiological changes without necessarily replacing the entire socket or modifying the physical socket structure. Adjustability has been stated as a desire for patients and as a potential solution to socket fit issues for several years, but market penetration for adjustable sockets in clinical prosthetics has been limited primarily due to lack of reimbursement.^2^ Adjustable-volume sockets present increased options for customization to the patient, present efficiencies during the fitting process, and allow for prolonged maintenance of fit compared to rigid-volume sockets.^13^ While there are several types of adjustable-volume sockets available on the market they are commonly omitted from literature reviews on socket design categorically.^2,3,14,15^ Therefore, the purpose of this article is to provide a market overview of adjustable-volume sockets and present their value proposition for end-users and potential providing clinics.

## MARKET OVERVIEW

**Figure 1:**
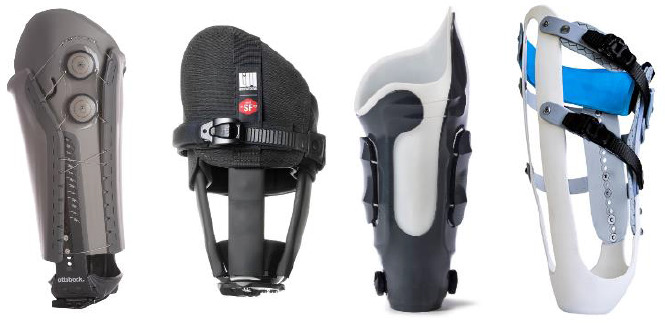
Examples of prefabricated, modular, custom with adjustable element, and custom-hybrid adjustable-volume sockets. Left to Right: Ottobock Varos, LIM Innovations Infinite TF, Click Medical RevoFit, Martin Bionics Socket-less Socket.

There are three major categories of adjustable-volume sockets available on the market today: prefabricated, modular, and custom sockets with adjustable elements (Figure 1). Prefabricated sockets are ordered by generic size from a supplier and individually fit to the patient by a skilled practitioner. These sockets must be individualized and fit in real-time with the patient and must be trimmed, bent, molded (with or without heat), padded, or otherwise modified resulting in alterations beyond minimal self-adjustment. Prefabricated sockets are often bivalve in design and utilize an adjustable closure mechanism such as a cable and dial, toggle latch, ratchet straps, hook and loop Velcro, or similar derivative. These sockets often incorporate locking or anatomical suspension. They may or may not have additional adjustable elements. Notable prefabricated sockets include the Varos socket from Ottobock [Duderstadt, Germany], Connect® TF from Össur [Reykjavic, Iceland], and the iFit Prosthetics [Pewaukee, WI, USA] system.

Modular sockets are ordered as a set of several prefabricated parts and assembled directly to the patient or a model of the patient's limb by a skilled practitioner. The process typically includes affixing generically-sized struts or panels to a common base with many possible configuration options. The struts or panels must be trimmed, bent, molded (with or without heat), or otherwise modified resulting in alterations beyond minimal self-adjustment to achieve an individual fit for the patient. Circumferential pressure and biomechanical control are then attained through some adjustable closure mechanism including ratchet straps, hook and loop Velcro, or similar derivative. They often use locking suspension but can be suspended via suction in some arrangements. The most notable modular system was the Infinite Socket™ line by LIM Innovations [San Francisco, CA, USA].^5^

The third form of adjustable sockets are custom sockets with adjustable elements. These fully-laminated sockets are fit to a model of a patient's limb with one or more adjustable elements added or fabricated in. These can include integrated adjustable features such as the tensioning cable with floating panel or ratchet straps which apply or release pressure to a cut-out or flexible portion of the socket. Addition of these adjustable socket features requires alterations in socket fabrication or disruption to the traditional physical structure of sockets. Notable adjustable socket features are the RevoFit™ by Click Medical [Steamboat Springs, CO, USA] and Quatro™ socket by Quorum Prosthetics [Windsor, CO, USA].^5^ Adjustable components are separately manufactured items added to the custom socket following the fitting process, or which alter the process only slightly. These components include air or fluid bladders such as the former Simbex [Lebanon, NH, USA] Active Contact System or Prosthetic Concepts [Little Rock, AR, USA] Pneu-fit™ system.^9^

An additional form of adjustable-volume socket, the custom-hybrid adjustable socket, contains major attributes of the previous three types. Custom-hybrid sockets differentiate themselves, - as they utilize some other radically-unique design aspects to provide micro-adjustability to the patient and macro-adjustability to the prosthetist. These sockets are justly custom as they can only be fabricated from a model of the patient's residual limb. They are hallmarked by removal of large portions of the conventional custom-laminated socket and replacement of foundational, key socket elements with truly flexible materials. Custom-hybrid designs alter traditional mechanical properties of rigid frames and flexible inner sockets in exchange for increased customization, flexibility, and adjustability. These adjustability options are presented both during and after the initial fitting process. The most notable custom-hybrid adjustable systems include the Socket-less™ Socket system by Martin Bionics [Oklahoma City, OK, USA] and the Sail socket by CJ Socket Technologies [Beverly, MA, USA].^5^

The Martin Bionics Socket-less Socket™ system replaces most of the conventional, rigid-volume socket with conforming materials which contour to the residual limb, providing a hammock-like fit. The inner socket is replaced with custom-configurable parts consisting of flexible, injection-molded plastic straps, adjustable webbing straps, thermoplastic and metal struts, and micro-adjustable closure mechanisms. These parts can be assembled in combination with each other to create a customized fit. The unique design of the Socket-less Socket™ allows for numerous configuration options for a wide variety of patient and limb types enabling macro-adjustability by the prosthetist and micro-adjustability by the patient. The Martin Bionics systems are available for all major amputation levels. All transfemoral (TF) applications utilize an adjustable SwingBrim™ which replaces the conventional rigid brim with a webbing-based conforming brim, thereby eliminating rigid contact at the ischial seat. A version called the Bikini Socket™, which utilizes Martin Bionics' Iliac Crest Stabilizers™ and ratchet closures, also exists for hip disarticulation and hemipelvectomy levels. The Socketless™ designs have many ancillary benefits reported by Martin Bionics including improved comfort, breathability, range of motion, restored muscular activation in the residual limb, and reduced heat retention compared to rigid-volume sockets.

Another custom-hybrid adjustable system is the CJ Sail Socket. This system replaces the traditional flexible inner socket with a textile Sail piece which has integrated Velcro closures. The sail is typically affixed to one side of a custom socket shell with rivets and the adjustable straps attach to the other side with chafes. The Sail socket is available for most major amputations levels and the TF systems are mostly sub-ischial.

## VALUE PROPOSITION

### End-Users

Adjustable-volume sockets are designed to accommodate a larger range of residual limb volumes from baseline than rigid-volume sockets.^13^ The solution to diurnal volume fluctuation in most traditional systems, which is typically in the form of limb volume loss with prolonged daily wear, is to add prosthetic socks to fill the resultant voids.^8,9^ This requires the user to fully remove the prosthesis which is inconvenient at best. This may occur several times daily resulting in substantial time lost. On the other hand, patients will often avoid going through the process of doffing the prosthesis to change socks to save time and convenience. This doffing avoidance exacerbates fit issues and can cause damage to the residual limb over time.

Options are more limited in rigid-volume sockets when the volume of the residual limb increases due to edema, weight gain, or some other physiological factor.^16^ Systems with a flexible inner socket and rigid frame can allow for the flexible inner socket to be removed to reveal an increased socket volume.^17^ Patients then add socks, or prosthetists can add pads, to restore fit intimacy. This situation is considered suboptimal, as the benefits of the flexible inner socket are lost and socket design is compromised, unless previously anticipated. Further, sockets are not always fabricated with a flexible inner socket or one that can be removed.

Since the volume accommodation strategies of adjustable-volume sockets are easier for the patient to make, typically through clothing or discretely, they are more likely to be implemented. This can lead to longer durations of optimal fit, increased wear time, and increased physical activity. Increased wear leads to more stable limb volumes and accelerated maturation as well.^8^ This means adjustable-volume sockets are not only able to accommodate residual limbs with frequent volume changes but may also reduce volume fluctuation over time. Therefore, adjustable-volume sockets present an opportunity for a short-term and long-term solution to volume fluctuation. Independently of this effect, adjustable-volume systems provide a potential solution to long-term volume fluctuation in that the socket volume can be adjusted to the user through adjustment of components or features without necessarily fabricating a new socket. Therefore, the patient would not have to commit to the time required for a replacement socket fitting and would reduce costs through reduced payments or co-insurance.

### Clinics

**Figure 2:**
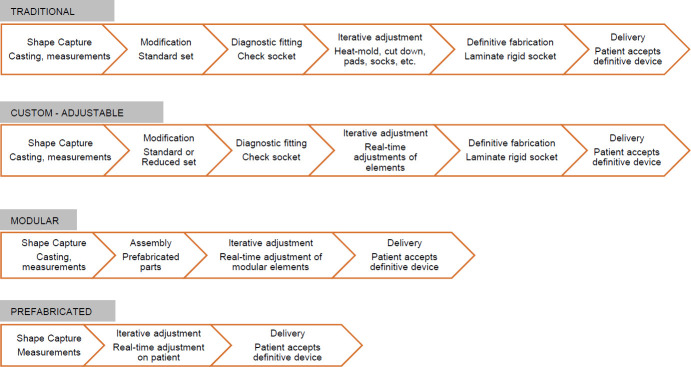
Socket Fitting Processes.

Rigid-volume sockets are currently fit through some form of residual limb shape capture, form modification, diagnostic fabrication, iterative alteration, and definitive fabrication (Figure 2).^18^ The shape capture portion is mainly accomplished through hand-casting-using plaster or fiberglass bandage, as well as three-dimensional scanning. Modification of the captured shape is accomplished manually to a physical model or digitally using a computer-aided design (CAD) program and are often standardized. Diagnostic fabrication includes creation and application of a clear plastic socket, commonly PETG, to the patient's residual limb to inspect fit and allow alterations.^4^ These alterations are made in an iterative fashion, sometimes with multiple check sockets, until a satisfactory fit is achieved. A definitive socket is then fabricated from the resultant form. Definitive sockets are designed for long-term use, with fewer options for alterations compared to the diagnostic sockets.

While this fabrication process is commonplace, rigid-volume sockets are inherently limited with some alterations being exceedingly difficult, time-consuming, or impossible to make through the various modification techniques and each having their associated costs. Some alterations are impossible to reverse once made, requiring the socket to be remade altogether. Therefore, the patient and practitioner are unavoidably presented with the decision to make the alteration or not. This results in a sub-optimal situation for both parties involved to achieve the best socket fit possible. Further, third-party payers often limit the quantity and frequency at which new sockets can be reimbursed. The reimbursement for periodic replacement of sockets often constitutes a significant percentage or majority of a prosthetic clinic's revenue stream.

Adjustable-volume sockets create efficiencies in - the fitting process by eliminating or simplifying steps. Prefabricated sockets are manufactured in mass quantities to create an economy of scale for the manufacturer. They are loosely designed around traditional socket designs and present one or several options for custom-fitting by the skilled practitioner. These options include pad kits, adjustable cables, and air bladders, while also enabling trimming and heat-molding.^13^ Prefabricated sockets eliminate the modification step and definitive fabrication portions of the process while simplifying diagnostic fabrication and iterative alteration into configuration of macro-adjustable elements in real-time with the patient. Shape capture is simplified to measurement for accurate size order. Prefabricated sockets present reasonable fit options to a majority of amputees who present with standard residual limb anatomy. Biomechanical control is a topic of debate with prefabricated sockets as associated transtibial (TT) designs implement pre-tibial pads to offload bony prominences and the TF designs are mostly sub-ischial in nature.

Modular sockets seek to create similar efficiencies in fitting by eliminating modification and simplifying diagnostic and definitive fabrication into assembly of prefabricated modules. An economy of scale can be realized in mass production of these modules similar to prefabricated sockets. Modular sockets present some advantages over rigid-volume sockets regarding customization as elements can be placed in direct response to residual limb anatomy and biomechanics. Many of the modules are themselves heat adjustable and can be individually customized in this way as well. Diagnostic sockets are sometimes used for offsite fabrication of these systems but are not always required. Modules often include component attachment blocks, struts, panels, and some circumferential binding element. Upper extremity designs can include cuffs and TF designs often implement a telescoping ischial seat module.^16,19^ These systems typically, but not always, include a micro-adjustable element.

Custom sockets with adjustable elements are varied in their fitting efficiencies. Many of the benefits with these systems come in the form of the aforementioned daily micro-adjustability for the patient. In custom sockets with integrated adjustable features, such as the cable/panel type, more fabrication time and labor are often required at the outset. With the RevoFit™ and Quatro™ systems, for example, the same standard fabrication techniques are required in addition to the feature installation.^5^ The increased socket fit range these systems present, however, may potentially reduce the number of diagnostic fitting and iterative changes required as they present additional options for customization and biomechanical control depending on application. Reduced number of follow-up appointments are often marketed by the system manufacturers. Custom sockets with adjustable components alter fabrication only slightly or not at all, but also present those options for adjustability in real-time with the patient.^9^

Custom-hybrid adjustable systems with multiple macro- and micro-adjustable attributes also present mixed efficiencies in fitting and follow-up.^5^ The Socket-less™ Socket systems by Martin Bionics simplify or eliminate several steps of the fabrication process including in shape capture, modification, and iterative alteration. Initial diagnostic and definitive fabrication are slightly prolonged by the additional assembly time needed to finish the increased topographical length of socket trimlines due to the many cut-outs, drilling holes to affix the Socket-less Socket™ components, and converting components from diagnostic to definitive. The total number of fitting appointments is typically reduced since iterative alterations are made in real-time with the patient versus in the lab and need for additional check sockets is often reduced. Customization of the Socket-less Socket™ includes simply moving a Chicago-type thumb screw from one hole to another in the thermoplastic straps. So, significant adjustments or socket re-makes can be reduced to a matter of seconds. Additionally, the adjustable nature of these sockets and options for customization throughout the product life cycle could facilitate reduced overutilization costs per beneficiary thereby reducing overall cost to third-party payers.

Shape capture and modification are simplified with the Martin Bionics system since large portions of the laminated socket are eliminated. The TF version, for example, eliminates the need for high-definition shape capture of the pelvic anatomy and modification of the traditional brim due to integration of the SwingBrim™. One approach of the TF system includes replacement of the laminated lateral wall with metal bars to suspend the components, presenting an efficiency by limiting shape capture and modification further to the distal third of the residual limb. The iterative alteration step is simplified as the check socket no longer requires heat guns and grinders but utilizes the real-time adjustments through straps with thumb screws instead. The macro-adjustability of the systems allows for considerably more options for customization to the patient and, perhaps more profoundly, the ability to reverse a socket adjustment if it is ultimately deemed undesirable to the patient.

The CJ sail socket is another custom-hybrid adjustable system. This system therefore presents efficiencies through simplification of the modification and iterative alteration steps of fabrication. Likewise, additional time may be required in diagnostic and definitive fabrication steps to prepare the socket and integrate the textile. Since the textile piece conforms to the patient's residual limb shape though, the need for extensive form modifications are reduced. The sub-ischial TF version also reduces the amount of shape capture detail and modification needed at the ischial seat. Structural testing of the CJ sail socket and Martin Bionics systems have not been disseminated in the literature to date, so long-term maintenance requirements are unknown compared to rigid-volume sockets which are known for durability. However, the major cause of socket replacement is from residual limb volume change and subsequent fit and discomfort issues which conforming and adjustable-volume sockets seek to resolve.

## CONCLUSION

The purpose of this article is to provide a market overview for the subcategory of adjustable prosthetic sockets, their reported end-user benefits, and potential fitting process efficiencies. The subcategories of pre-fabricated, modular, and custom sockets with adjustable elements are identified. The custom sockets with adjustable elements subcategory is further delineated into addition of adjustable component or adjustable features. Custom-hybrid adjustable systems, which span all three major categories and have some other radically-unique design feature, are also presented. Adjustable sockets present opportunities for efficiencies through innovation of the various stages of fitting including shape capture, form modification, diagnostic fabrication, iterative alteration, and definitive fabrication. Long-term efficiencies are also created through reported reductions in follow-up time and overutilization. Adjustable sockets in their various forms are disruptive technologies, but likely represent a lasting innovation to the field of clinical prosthetics.

## CALL TO ACTION

### Reimbursement Reform

Adjustable-volume socket technologies have disrupted the traditional delivery model in clinical prosthetics and reimbursement reform is needed to ensure longevity and capitalization of the trend. Since the technology shifts the emphasis in fitting from skilled labor of fabrication to clinical expertise and long-term care, corresponding reimbursement items should be introduced. In the current fee-for-device delivery model this would manifest itself in the form of additional L-codes. In a hybridized model, allowances for clinical services not associated with initial delivery of the device should be implemented including evaluation, outcome measure collection, and long-term adjustment.^20^ In addition, the administrative burden for repair and replacement of minor parts billing should be reduced to encourage their use. These proposed reforms should ultimately reduce cost for third-party payers and the healthcare system overall through reduced costs per beneficiary resultant from reduced overutilization.

The principles of these adjustable-volume systems also incorporate well into fee-for-outcome and fee-for-value models through the presented efficiencies and prospect of improved outcomes in the long-term. These reimbursement models have permeated allied health in other areas, but not durable medical equipment yet. Prosthetic services should be separated from DME in the policy of payers, as it is in the Uniform Glossary of Health Coverage and Medical Terms, establishing the field as its own independent specialty.^21^ This would allow for simplified implementation of cost-saving strategies apart from fee-for-device including fee-for-outcome, bundled payments, and capitation.

### Prosthetist Adaptations

As innovation in the field of clinical prosthetics occurs and reimbursement models evolve in the current progressive healthcare climate, prosthetists must also advance care delivery systems. Currently, prosthetic prescription, fitting, and delivery relies heavily on the individual experience and expertise of the clinicians. However, the experience and educational background of these professionals is quite varied, reflecting the changing collective thought of the field at various points in time. If prosthetists are to set themselves apart from durable medical equipment suppliers, their associated skillset must also set itself apart. Since the patient population of prosthetic users is so unique, this skillset must include an unparalleled and self-evident expertise of the most unique aspect of the patient: the residual limb. Further acceptance of advanced assessment techniques, such as digital shape capture, activity monitoring, and physical performance outcome measures, is required. In addition, the field of prosthetics should continue and more earnestly push for licensure of its practitioners throughout the United States, Canada, and abroad. This will enable the true clinical independence, professional validation, and service-related reimbursement currently being sought. Other allied health professionals including physical therapists, occupational therapists, podiatrists, and audiologists have accomplished similar goals correspondingly.

## DECLARATION OF CONFLICTING INTERESTS

Tyler D. Klenow is an employee of Martin Bionics Clinical Care and Joel Schulz is an employee for Martin Bionics Innovations, the providers of the Socket-less™ Socket systems.

## SOURCES OF SUPPORT

None.

## AUTHOR SCIENTIFIC BIOGRAPHY

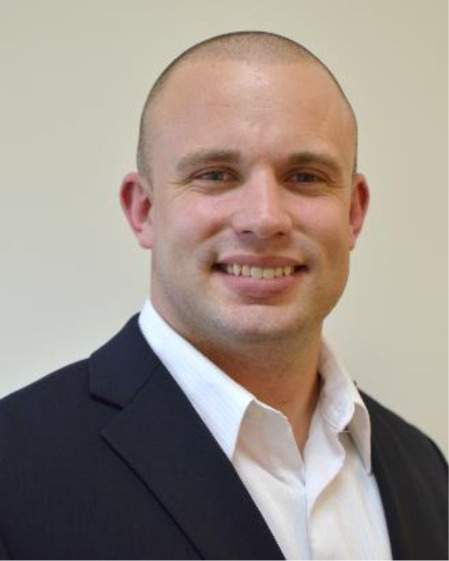



**Tyler Klenow**, MS, MBA, LCPO, FAAOP is a graduate of the Master of Science in Orthotics & Prosthetics program at Eastern Michigan University and the Master of Business Administration program in the Nathan M. Bisk School of Business at Florida Institute of Technology. He is currently the co-chair of the clinical education planning committee for the American Orthotic & Prosthetic Association (AOPA) and has previously served as the Chairman of the Outcomes Research Committee for the American Academy of Orthotists and Prosthetists. Tyler specializes in prosthetic research including outcome measures, biomechanics, and secondary knowledge. He joined Martin Bionics Clinical Care as a Program Manager and Clinic Leader in 2020.

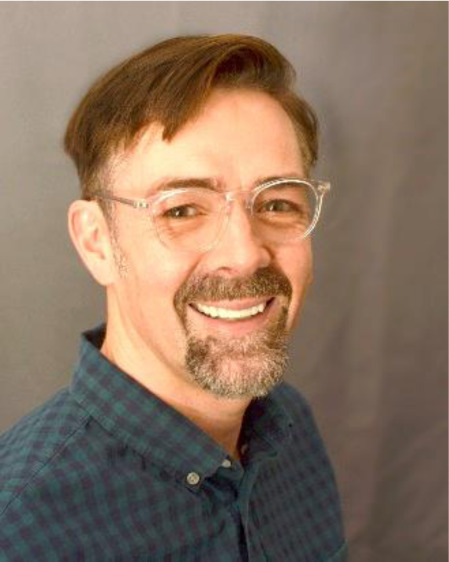



**Joel Schulz,** BA, BSPO, CLP is a graduate of the Prosthetics and Orthotics program at the University of Washington and has worked in several roles in the prosthetics industry over an 18-year career. He worked on the The Defense Advanced Research Projects Agency (DARPA) Revolutionizing Prosthetics 2009 Project with Johns Hopkins Applied Physics Laboratory while at Orthocare Innovations. Joel co-developed a fabric-based exoskeletal jacket with the National Aeronautics and Space Administration Warrior Web program. He has experience in computer aided design (CAD), animatronics, prosthetic socket design, and has lectured for prosthetic manufacturers and training programs as well. He worked for the original Martin Bionics company in 2007 and joined Martin Bionics Innovations as a design engineer and research prosthetist in 2014.
